# Performance of Positron Emission Tomography and Positron Emission Tomography/Computed Tomography Using Fluorine-18-Fluorodeoxyglucose for the Diagnosis, Staging, and Recurrence Assessment of Bone Sarcoma: A Systematic Review and Meta-Analysis

**DOI:** 10.1097/MD.0000000000001462

**Published:** 2015-09-11

**Authors:** Fanxiao Liu, Qingyu Zhang, Dezhi Zhu, Zhenfeng Li, Jianmin Li, Boim Wang, Dongsheng Zhou, Jinlei Dong

**Affiliations:** From the Department of Orthopedics, Provincial Hospital Affiliated to Shandong University, Jinan, Shandong, China (FL, BW, DZ); Department of Orthopedics, Qilu Hospital, Shandong University, Jinan, Shandong, China (QZ, ZL, JL); and Department of Orthopedics, Heze Peony People's Hospital, Heze, Shandong, China (DZ, FL).

## Abstract

To investigate the performance of fluorine-18-fluorodeoxyglucose (^18^F-FDG) positron emission tomography (PET) and PET/computed tomography (CT) in the diagnosis, staging, restaging, and recurrence surveillance of bone sarcoma by systematically reviewing and meta-analyzing the published literature.

To retrieve eligible studies, we searched the MEDLINE, Embase, and the Cochrane Central library databases using combinations of following Keywords: “positron emission tomography” or “PET,” and “bone tumor” or “bone sarcoma” or “sarcoma.” Bibliographies from relevant articles were also screened manually. Data were extracted and the pooled sensitivity, specificity, and diagnostic odds ratio (DOR), on an examination-based or lesion-based level, were calculated to appraise the diagnostic accuracy of ^18^F-FDG PET and PET/CT. All statistical analyses were performed using Meta-Disc 1.4.

Forty-two trials were eligible. The pooled sensitivity and specificity of PET/CT to differentiate primary bone sarcomas from benign lesions were 96% (95% confidence interval [CI], 93–98) and 79% (95% CI, 63–90), respectively. For detecting recurrence, the pooled results on an examination-based level were sensitivity 92% (95% CI, 85–97), specificity 93% (95% CI, 88–96), positive likelihood ratio (PLR) 10.26 (95% CI, 5.99–17.60), and negative likelihood ratio (NLR) 0.11 (95% CI, 0.05–0.22). For detecting distant metastasis, the pooled results on a lesion-based level were sensitivity 90% (95% CI, 86–93), specificity 85% (95% CI, 81–87), PLR 5.16 (95% CI, 2.37–11.25), and NLR 0.15 (95% CI, 0.11–0.20). The accuracies of PET/CT for detecting local recurrence, lung metastasis, and bone metastasis were satisfactory. Pooled outcome estimates of ^18^F-FDG PET were less complete compared with those of PET/CT.

^18^F-FDG PET and PET/CT showed a high sensitivity for diagnosing primary bone sarcoma. Moreover, PET/CT demonstrated excellent accuracy for the staging, restaging, and recurrence surveillance of bone sarcoma. However, to avoid misdiagnosis, pathological examination or long-term follow-up should be carried out for ^18^F-FDG-avid lesions in patients with suspected bone sarcoma.

## INTRODUCTION

In human neoplasms, primary bone sarcoma is a rare entity, among which, osteosarcoma ranks as the most common histological type, followed by chondrosarcoma, Ewing sarcoma, chordoma, malignant fibrous histiocytoma, angiosarcoma, and others. According to a large report, the former 5 types account for >90% of all bone sarcomas.^1^ The incidence of osteosarcoma peaks in the second decade of life, with a second peak occurring in patients >60 years old.^2^ Although the 5-year overall survival of bone sarcoma has improved greatly with the introduction of pre and postoperative chemotherapy and with advances in surgical techniques, the prognosis of patients with local recurrence or distant metastasis remains unfavorable.^3–6^ Therefore, stratifying high-risk patients at an early stage or during follow-up plays a crucial role for implementing appropriate treatment strategies.

Diagnostic imaging provides information concerning the appearance, extent, and radiographical characteristics of bone lesions, contributing significantly to the diagnosis and prognosis of the disease.^7^ Morphological imaging modalities such as plain film, computed tomography (CT), and magnetic resonance imaging (MRI) are all commonly used to assess bone sarcoma. In addition, fluorine-18-fluorodeoxyglucose positron emission tomography (^18^F-FDG PET) can be used to quantify the physiological activity of bone sarcomas, denoted by increased glucose uptake, which leads to biochemical changes before the onset of anatomic changes.^8,9^ More recently, the incorporation of CT-derived morphological information with traditional ^18^F-FDG PET has further improved the diagnostic performance of imaging techniques. Presently, ^18^F-FDG PET and PET/CT have been broadly applied for diagnosis, biopsy guidance, and chemotherapy response evaluation in a variety of solid tumors, including lung cancer, cervical cancer, and pancreatic carcinoma.^10–14^

Multiple trials have investigated the value of ^18^F-FDG PET and PET/CT for the diagnosis, staging, and recurrence detection of bone sarcoma, but the results have been inconclusive. However, most of those trials analyzed a small number of patients, which weakened their power and reliability. A 2004 meta-analysis^15^ reported a sensitivity of 91% and a specificity of 85% for ^18^F-FDG PET for the differentiation of bone and soft-tissue sarcomas from benign lesions. However, this investigation was not specially aimed at bone sarcomas and did not appraise the utility of ^18^F-FDG PET comprehensively. Presently, ^18^F-FDG PET or PET/CT are not regarded as a routine procedures in the management algorithm of bone sarcomas. To obtain a more precise conclusion on the utility of ^18^F-FDG PET or PET/CT for the management of bone sarcoma, we searched the published literature and conducted a systematic review and meta-analysis.

## METHODS

### Search Strategy

A systematic electronic search of MEDLINE, Embase, and Cochrane Library databases was conducted to select relevant articles. We used combinations of following keywords: “PET” or “positron emission tomography,” and “bone tumor” or “bone sarcoma” or “sarcoma.” The search process was last updated on May 1, 2015 without language limitations. The bibliographies of pertinent articles (meta-analysis, reviews, editorials, and trials) and guidelines were also screened manually to retrieve additional eligible studies.

### Study Selection

Eligible studies for this meta-analysis had to meet following criteria: clinical studies; diagnosis, staging, restaging, or recurrence surveillance performance of ^18^F-FDG PET or PET/CT in participants with primary bone sarcoma; definite outcome confirmed with trustworthy reference tests (histopathological examination or follow-up); all participants were human; ^18^F-FDG was administered intravenously as tracer. Exclusion criteria included case reports or trials evaluating <5 patients with bone sarcoma; reviews, editorials, meta-analyses, letters, comments, and other nonoriginal articles; and congress proceedings, because of the lack of necessary information. If ≥2 articles contained overlapping data, the 1 with the most comprehensive data or that was published most recently was included in the quantitative analysis.

Three investigators (FL, QZ, and ZL) independently evaluated retrieved articles. Any disagreements were resolved by discussion and consensus.

### Data Extraction

Data retrieved from eligible studies included: study-related information: first author's surname, year of publication, country of origin, and study design; patient-related data: number and participants, age, and sex; technical details: ^18^F-FDG PET or PET/CT, injection dose, injection-to-measure interval, methods of image analysis, and reference tests; accuracy data: the number of true positive (TP), false positive (FP), true negative (TN), and false negative (FN) cases on a per examination-based or lesion-based level (extracted directly or recalculated if necessary). To avoid bias, this process was conducted by 2 reviewers (FL and QZ) independently and checked repeatedly.

### Quality Assessments

The methodological quality of eligible studies was estimated using the quality assessment tool for diagnostic accuracy studies (QUADAS).^16^ This system is composed of 14 items including the patient spectrum covered, reference standards, test execution, study withdrawals, indeterminate results as well as verification, review, clinical review, incorporation, and disease progression biases. A 1-point score was given for each item and studies with high scores were considered as good reports.

### Statistical Methods

For individual studies, we recalculated the sensitivity, specificity, positive likelihood ratio (PLR), negative likelihood ratio (NLR), and diagnostic odds ratio (DOR) (with 95% confidence interval [CI]) of ^18^F-FDG PET or PET/CT for the diagnosis, staging, restaging, and recurrence surveillance of bone sarcoma on examination-based or lesion-based level. We visualized the summary receiver operating characteristic (sROC) curve to see if there is threshold effect. If a threshold effect was not found, the random-effect model was applied to pool outcome estimates. Otherwise, diagnostic accuracy was assessed using the Q∗-index and the area under the sROC (AUC). Subgroup analyses were performed according to metastases locations, recurrence, and the modality used (^18^F-FDG PET or PET/CT). All statistical analyses were conducted using Meta-Disc software 1.4.

Because data were extracted from published literature, informed consent or ethical approval was not required for this study. This study conformed to the standardized items described by “the Preferred Reporting Items for Systematic Reviews and Meta-Analyses (PRISMA)” statement.^17^

## RESULTS

### Eligible Studies

**FIGURE 1:**
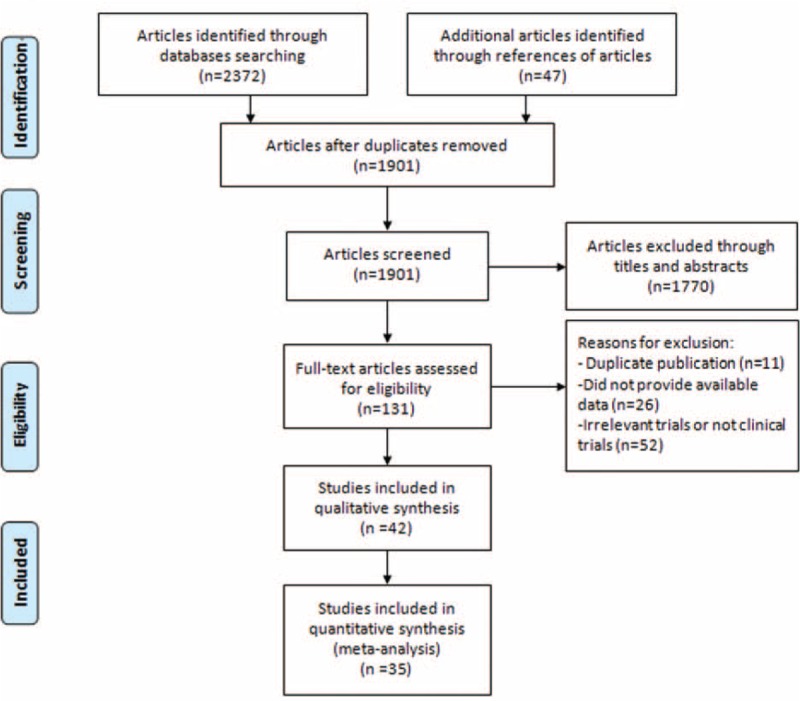
Selection flow chart for studies included in the systematic review and meta-analysis.

During database and bibliography searches, 1901 relevant articles were identified. We firstly excluded 1770 ineligible articles by browsing titles and abstracts. Subsequently, the remaining ones were downloaded and reviewed as full-text versions. Eventually, 42 articles published between 1991 and 2015 were included in our investigation, among which 19^18–36^ evaluated bone sarcomas using ^18^F-FDG PET, whereas 24^36–59^ used PET/CT. The article searching process and exclusion criteria are shown in Figure 1.

Of the 42 articles, 35^19–24,27–29,32–34,36–52,54–59^ provided enough data to recalculate sensitivity and specificity and were included in the quantitative analysis, whereas the remaining 7^18,25,26,30,31,35,53^ were analyzed qualitatively. One article was published in Chinese^45^ and the remainders were published in English. Lesions were classified by ^18^F-FDG status according to the methods and cutoffs defined in individual trials. Although several studies included overlapping patients, they presented different data concerning subgroup analysis. For methodological quality according to QUADAS, 9 studies achieved 13 points, 6 studies achieved 12, 18 achieved 11, 3 achieved 10, and 6 achieved 9. The detailed information of included studies and extracted data are presented in Tables 1–3 .

**TABLE 1:**
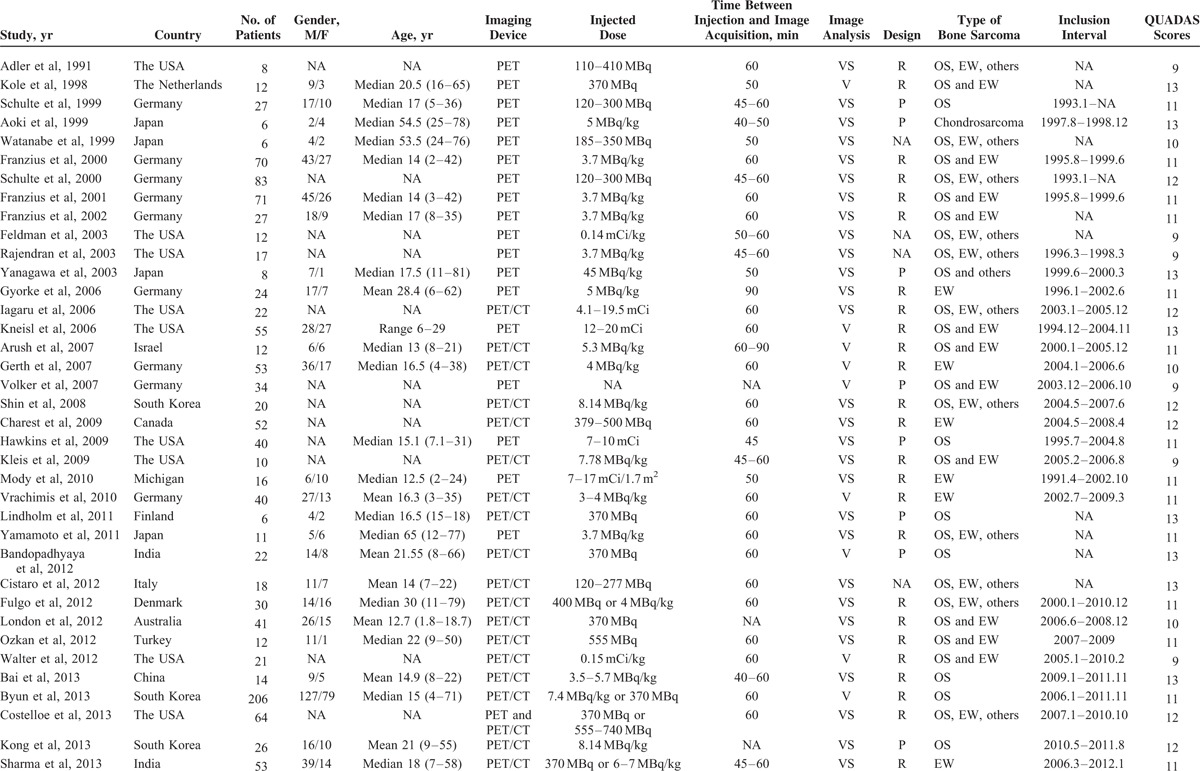
Main Characteristics of the Included Studies in the Systematic Review and Meta-Analysis

**TABLE 1 (Continued):**
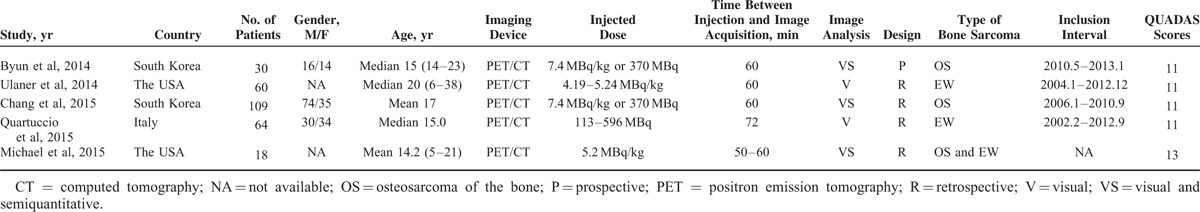
Main Characteristics of the Included Studies in the Systematic Review and Meta-Analysis

**TABLE 2:**
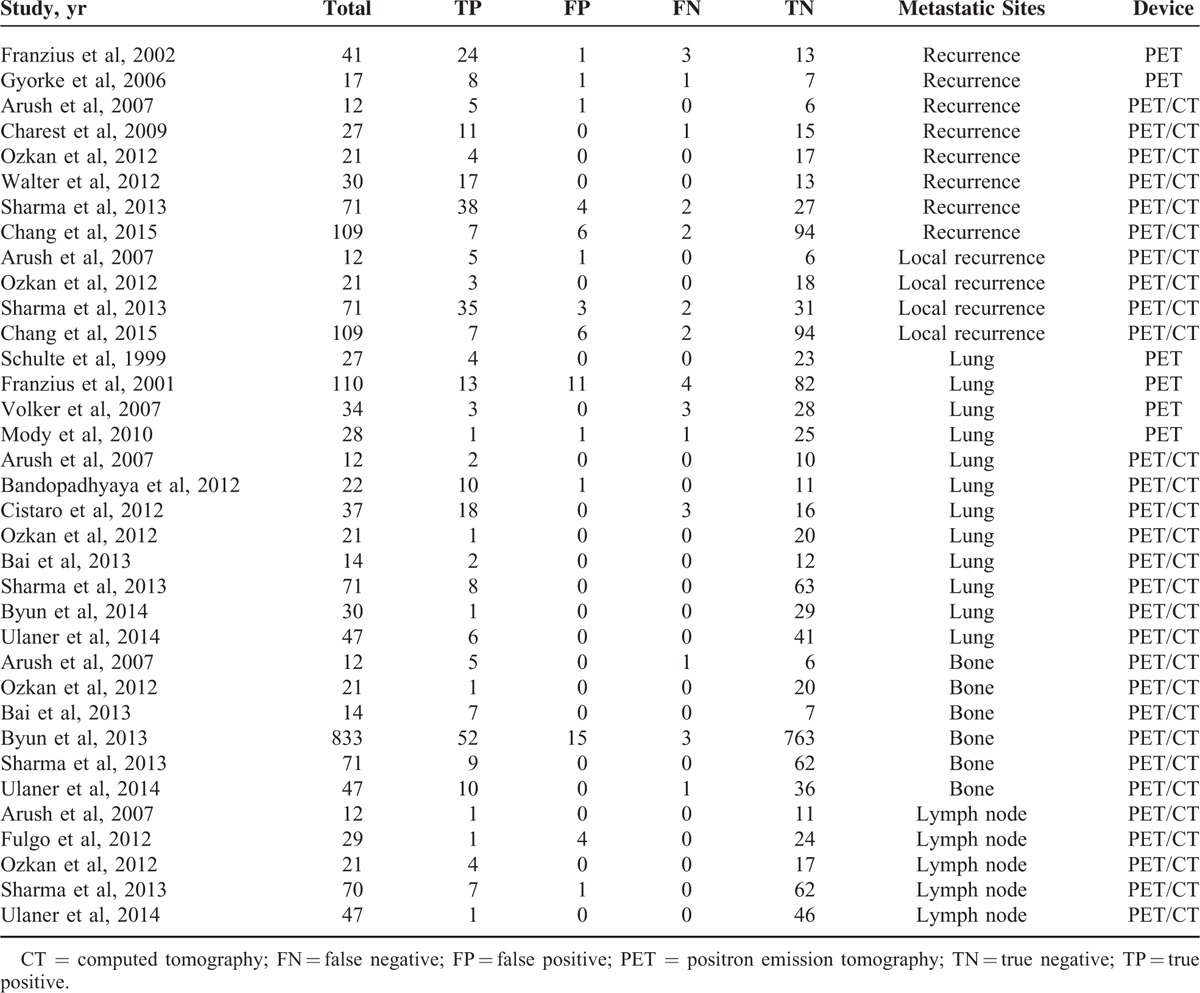
Diagnostic Accuracy of PET/CT and PET on a Lesion-Based Analysis

**TABLE 3:**
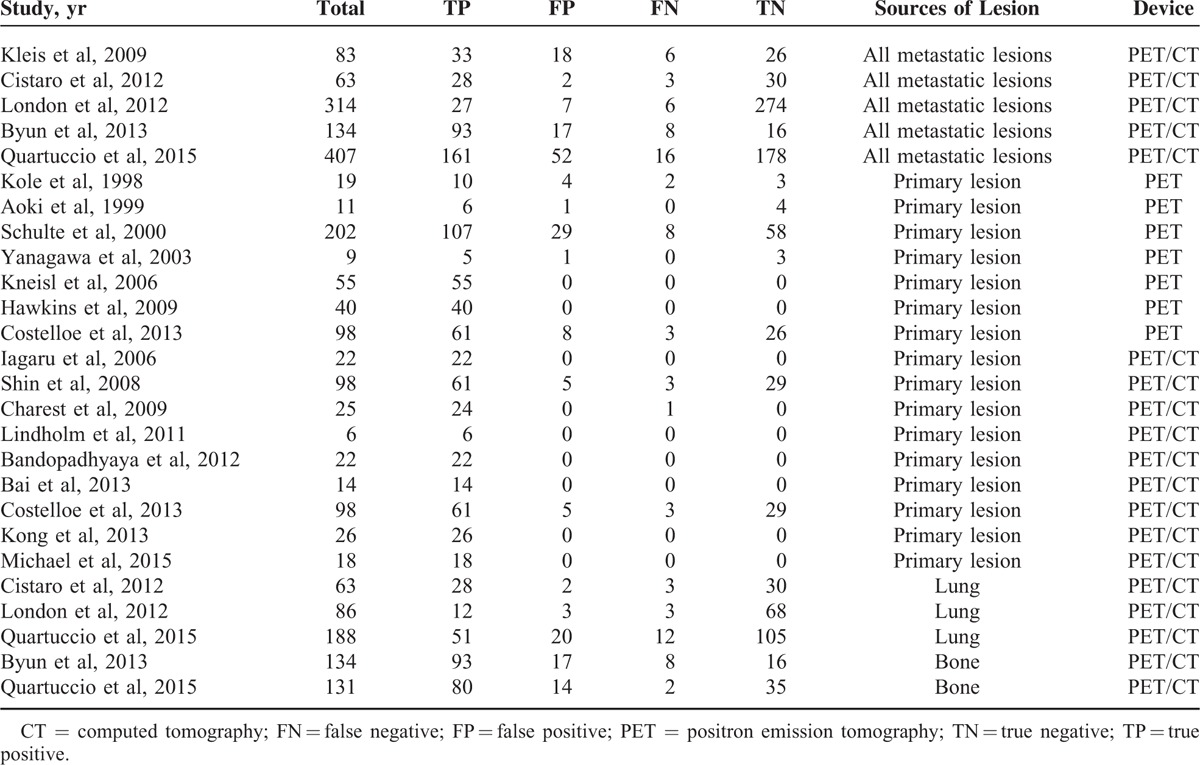
Diagnostic Accuracy of PET/CT and PET on an Examination-Based Analysis

### Differentiation of Primary Bone Sarcoma From Benign Lesions

**FIGURE 2:**
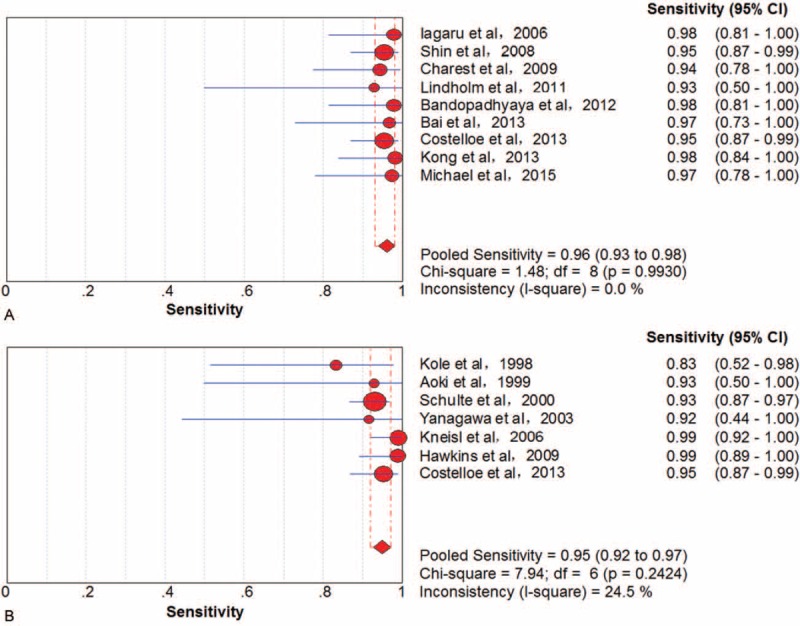
Performance of ^18^F-FDG PET/CT and PET for the diagnosis of primary bone sarcomas on a lesion-based analysis: (A) pooled sensitivity of ^18^F-FDG PET/CT and (B) pooled sensitivity of PET. ^18^F-FDG = fluorine-18-fluorodeoxyglucose; CT = computed tomography; PET = positron emission tomography.

Nine studies^36,39,43,45,51,52,54,56,59^ involving 251 patients investigated the performance of PET/CT to differentiate primary bone sarcomas from benign bone diseases. On a lesion-based level, there was no threshold effect. The pooled sensitivity and specificity were 96% (95% CI, 93–98) (Figure 2A) and 79% (95% CI, 63–90), respectively. There was no significant between-study heterogeneity for included outcome estimates (all I^2^ = 0).

Seven studies^20,22,24,29,32,34,36^ involving 434 patients described the ability of ^18^F-FDG PET to differentiation bone sarcomas from benign lesions. There was no threshold effect in lesion-based data. The pooled sensitivity and specificity were 95% (95% CI, 92–97) (Figure 2B) and 68% (95% CI, 60–76), respectively. There was no significant between-study heterogeneity for included outcome estimates (I^2^ = 0 and 24.5%, respectively).

### Recurrence

**FIGURE 3:**
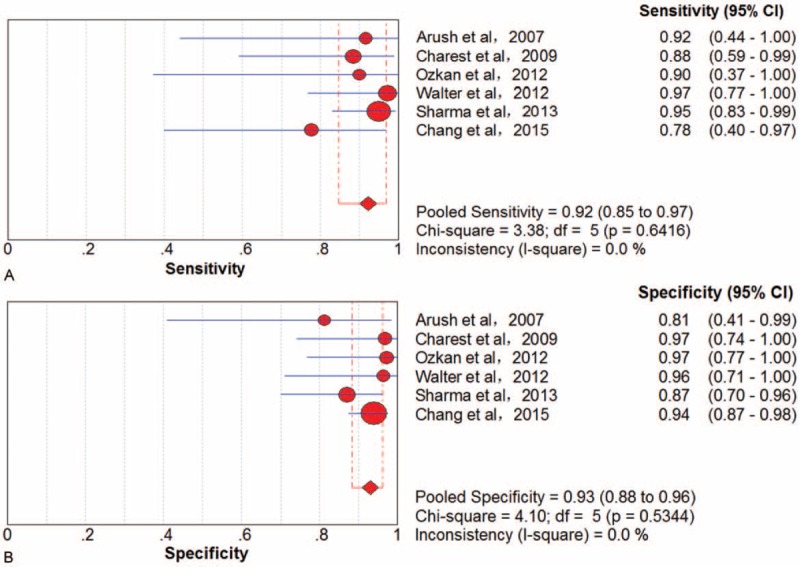
Performance of ^18^F-FDG PET/CT to detect recurrence of bone sarcomas on an examination-based analysis: (A) pooled sensitivity and (B) pooled specificity. ^18^F-FDG = fluorine-18-fluorodeoxyglucose; CT = computed tomography; PET = positron emission tomography.

Six trials^38,42,47,48,54,57^ involving 270 examinations addressed bone sarcoma recurrence using ^18^F-FDG PET/CT. There was no threshold effect in examination-based data. The pooled results for ^18^F-FDG PET to detect recurrence indicated that the sensitivity was 92% (95% CI, 85–97), specificity was 93% (95% CI, 88–96), PLR was 10.26 (95% CI, 5.99–17.60), NLR was 0.11 (95% CI, 0.05–0.22), and DOR was 113.12 (95% CI, 40.34–317.26) (Figure 3). There was no significant between-study heterogeneity for included outcome estimates (all I^2^ = 0).

Two trials^23,27^ involving 58 examinations addressed bone sarcoma recurrence using ^18^F-FDG PET. The pooled results for ^18^F-FDG PET to detect recurrence indicated that sensitivity was 89% (95% CI, 74–97, specificity was 91% (95% CI, 71–99), NLR was 9.34 (95% CI, 2.49–35.06), PLR was 0.12 (95% CI, 0.05–0.31), and DOR was 81.68 (95% CI, 12.92–516.36). There was no significant between-study heterogeneity for included outcome estimates (all I^2^ = 0). The sROC was unavailable because of the limited number of studies.

#### Local Recurrence

**FIGURE 4:**
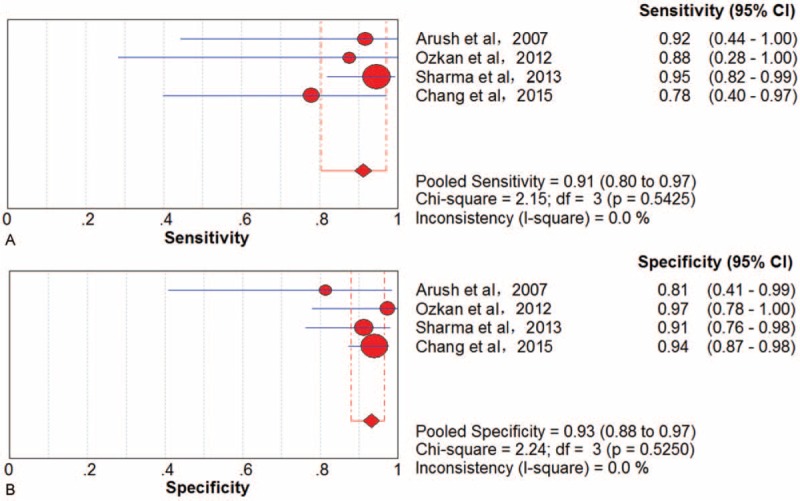
Performance of ^18^F-FDG PET/CT to detect local recurrence of bone sarcomas on an examination-based analysis: (A) pooled sensitivity and (B) pooled specificity. ^18^F-FDG = fluorine-18-fluorodeoxyglucose; CT = computed tomography; PET = positron emission tomography.

Four trials^38,42,48,57^ involving 213 examinations addressed local bone sarcoma recurrence using ^18^F-FDG PET/CT. There was no threshold effect in examination-based data. The pooled results for ^18^F-FDG PET to detect local recurrence were sensitivity 91% (95% CI, 80–97), specificity 93% (95% CI, 88–97), PLR 10.89 (95% CI, 6.01–19.72), NLR 0.12 (95% CI, 0.06–0.28), and DOR 96.69 (95% CI, 30.59–305.59) (Figure 4). There was no significant between-study heterogeneity for included outcome estimates (all I^2^ = 0).

There were no studies addressing local recurrence of bone sarcoma using ^18^F-FDG PET.

### Distant Metastasis

**FIGURE 5:**
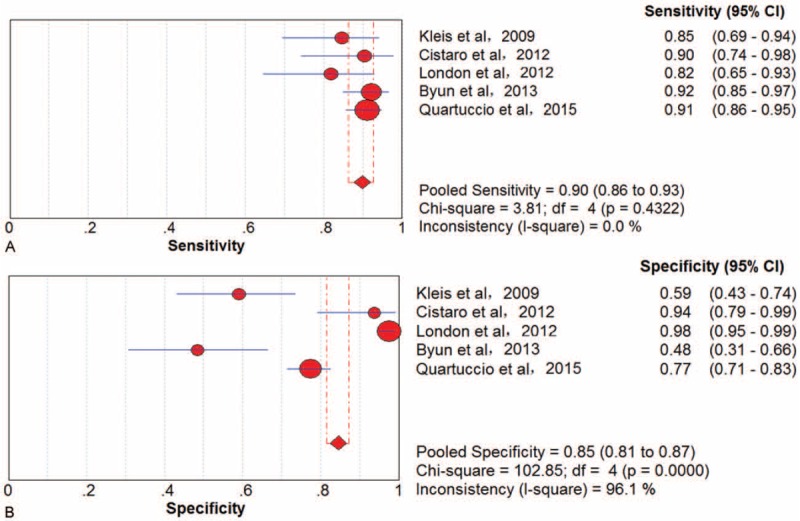
Performance of ^18^F-FDG PET/CT to detect distant metastasis of bone sarcomas on a lesion-based analysis: (A) pooled sensitivity and (B) pooled specificity. ^18^F-FDG = fluorine-18-fluorodeoxyglucose; CT = computed tomography; PET = positron emission tomography.

Five trials^37,44,46,50,55^ involving 1001 lesions were available. There was no threshold effect in lesion-based data. The pooled results for ^18^F-FDG PET to detect distant metastatic lesions of bone sarcoma were sensitivity 90% (95% CI, 86–93), specificity 85% (95% CI, 81–87), PLR 5.16 (95% CI, 2.37–11.25), NLR 0.15 (95% CI, 0.11–0.20), and DOR 33.87 (95% CI, 11.50–99.77) (Figure 5). There was significant between-study heterogeneity for specificity, PLR, and DOR (I^2^ = 96.1%, 93.8%, and 81.7%, respectively).

On a lesion-based level, 1 study^23^ involving 163 lesions was available to analyze distant metastasis of bone sarcoma using ^18^F-FDG PET. The sensitivity and specificity were 85% and 78%, respectively.

#### Lung Metastasis

**FIGURE 6:**
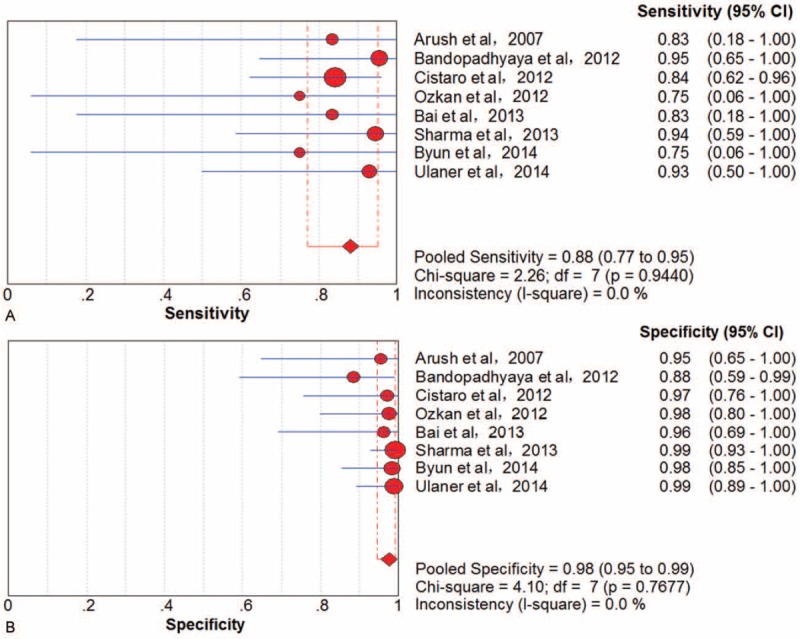
Performance of ^18^F-FDG PET/CT to detect lung metastasis of bone sarcomas on an examination-based analysis: (A) pooled sensitivity and (B) pooled specificity. ^18^F-FDG = fluorine-18-fluorodeoxyglucose; CT = computed tomography; PET = positron emission tomography.

Eight trials^40–42,45,48,50,51,57^ involving 254 examinations addressed lung metastasis of bone sarcoma using ^18^F-FDG PET/CT. There was no threshold effect in examination-based data. The pooled results for ^18^F-FDG PET to detect lung metastasis were sensitivity 88% (95% CI, 77–95), specificity 98% (95% CI, 95–99), PLR 23.71 (95% CI, 10.00–56.23), NLR 0.15 (95% CI, 0.07–0.29), and DOR 249.48 (95% CI, 64.91–958.81) (Figure 6). There was no significant between-study heterogeneity for included outcome estimates (all I^2^ = 0).

For lesion-based analysis of ^18^F-FDG PET/CT, 3 trials^37,46,50^ involving 337 lesions were available. There was no threshold effect in lesion-based data. The pooled results were sensitivity 83% (95% CI, 75–90), specificity 89% (95% CI, 84–93), PLR 9.75 (95% CI, 3.67–25.92), NLR 0.20 (95% CI, 0.13–0.30), and DOR 52.05 (95% CI, 15.17–178.60).

Four trials^19,21,28,33^ involving 58 examinations addressed lung metastasis using ^18^F-FDG PET. There was no threshold effect in examination-based data. The pooled results for ^18^F-FDG PET to detect lung metastasis were sensitivity 71% (95% CI, 52–86), specificity 92% (95% CI, 87–96), PLR 8.78 (95% CI, 4.11–18.76), NLR 0.38 (95% CI, 0.23–0.64), and DOR 32.98 (95% CI, 11.16–97.45). There was significant between-study heterogeneity for specificity (I^2^ = 51.1%). A lesion-based analysis could not be performed because of lack of data.

#### Bone Metastasis

Six trials^40,42,44,45,48,57^ involving 998 examinations addressed bone metastasis of bone sarcoma using ^18^F-FDG PET/CT on an examination-based level. There was no threshold effect in examination-based data. The pooled results for ^18^F-FDG PET to detect bone metastasis were sensitivity 92% (95% CI, 85–97), specificity 98% (95% CI, 97–99), PLR 46.23 (95% CI, 28.97–73.77), NLR 0.10 (95% CI, 0.05–0.20), and DOR 566.19 (95% CI, 206.02–1556.04). There was no significant between-study heterogeneity for included outcome estimates (all I^2^ = 0).

Two trials^37,44^ involving 265 lesions investigated ^18^F-FDG PET/CT on a lesion-based level. The pooled results for ^18^F-FDG PET/CT to detect bone metastases were sensitivity 95% (95% CI, 90–97), specificity 62% (95% CI, 51–73), PLR 2.43 (95% CI, 1.26–4.67), NLR 0.08 (95% CI, 0.01–0.46), and DOR 30.64 (95% CI, 3.34–281.48).

A single study^30^ was available to analyze the diagnostic accuracy of ^18^F-FDG PET for detecting bone metastasis of bone sarcoma. The sensitivity was 80% on a lesion-based level.

#### Lymph Node Metastasis

Five studies^40,42,48,49,57^ used PET/CT on an examination-based level. These studies presented a total of 14 TP cases and no FN cases. The specificity was 96% (95% CI, 91–98). However, because lymph node metastases occur rarely in patients with bone sarcoma, these results should be interpreted cautiously.

## DISCUSSION

Multiple studies have attempted to investigate the performance of ^18^F-FDG PET and PET/CT as noninvasive diagnostic tools for bone sarcomas, but the results have been heterogeneous. By performing a systemic review and meta-analysis of the published data, we could safely suggest that PET/CT is a useful tool for the diagnosis, staging, restaging, and recurrence surveillance of bone sarcoma.

Bone sarcomas have an elevated rate of glycolysis. After intravenously injection, fluorine-18-fluorodeoxygucose (^18^F-FDG), a radioactive analogue of glucose, accumulates in malignant cells. By detecting lesions with high uptake of this tracer, ^18^F-FDG PET and PET/CT have been utilized for several aspects of bone sarcoma assessment. For example, ^18^F-FDG uptake in different tumor areas is closely correlated to biological aggressiveness and histological grade; therefore, taking biopsies from maximum uptake regions improves the diagnostic success rate.^60^ In addition, standardized uptake value before (SUV1) and after (SUV2) chemotherapy can be suggestive of histological response. A previous meta-analysis of osteosarcoma^61^ revealed that an SUV2:1 ratio of <0.5 or an SUV2 of <2.5 significantly predicted tumor necrosis, whereas >90% decrease of metabolic sarcoma volume was sought for Ewing sarcomas.^62^

Functional imaging of primary lesions to determine local extent and soft-tissue involvement is performed as an adjuvant to MRI. In 1996, Dehdashti et al^63^ first described the ability of ^18^F-FDG PET to differentiate bone malignancies from benign lesions. When using a SUVmax cut-off of 2.0, the sensitivity and specificity were 93% and 80%, respectively. Subsequent studies supported their findings. FDG uptake can also provide valuable information for histological grading of musculoskeletal sarcoma. However, in the present study, the specificities of ^18^F-FDG PET and PET/CT for differentiating malignant and benign bone lesions and for determining histological grade were not satisfactory because overlapping SUVmax values were observed for several histological subtypes and grades of malignant and benign bone lesions.^60^ Therefore, although ^18^F-FDG PET and PET/CT possessed a high sensitivity for identifying primary bone sarcomas, they could not replace histopathological examination as the gold standard for initial grading. However, after the initial diagnosis, ^18^F-FDG PET and PET/CT could be used for whole-body staging and recurrence surveillance.

Bone sarcoma metastasis to distant sites can result in unfavorable survival outcomes. According to the published data, the lung was the most commonly involved site, closely followed by “other” bone sites, whereas lymph node and soft-tissue metastases rarely occurred.^64^ Because the early management of metastatic lesions could improve survival, initial staging and timely restaging during follow-up are indispensable. Compared with other imaging modalities, a major advantage of ^18^F-FDG PET and PET/CT is the ability to assess systemic metastases. We found that the performance of ^18^F-FDG PET/CT in detecting metastases was excellent. However, in the sub-group analysis, the performance of PET/CT in detecting lung metastases was not as good as that for detecting “other” bone metastases on a lesion-based level. In addition, the subgroup analysis revealed that the sensitivity of ^18^F-FDG PET for identifying metastases on the examination-based level was unsatisfactory (71%). The discrepancies in subgroup analyses could be explained by the size of the metastatic nodules at specific sites, which might influence the data. CT imaging is usually performed at low resolution and conducted during shallow breathing. In addition, because of the partial volume effect caused by respiratory activities, the recorded SUV normally dwindles. Iagaru et al^59^ examined 106 bone and soft-tissue sarcomas, and the FN rates for lung metastases were significantly higher in patients with subcentimeter nodules. Furthermore, Cistaro et al^50^ evaluated 18 bone sarcomas and did not find any significance of the SUVmax or SUV ratio for the appraisement of lung nodules <6 mm in size. The survival of bone sarcoma patients with bone-plus-lung or even bone-only metastases is poorer than those with lung-only metastasis.^4^ Bone scintigraphy is another commonly used whole-body modality to detect bone metastases. In 2000, Franzius et al^30^ compared the performance of ^18^F-FDG PET and bone scintigraphy for the detection of bone metastasis. They suggested that bone scintigraphy was superior to ^18^F-FDG PET. However, more recently, several trials^40,44^ have suggested that, compared with bone scintigraphy, PET/CT demonstrated better accuracy for detecting bone metastases. In agreement, the present meta-analysis revealed remarkable sensitivity and specificity of PET/CT for the detection of bone metastases, suggesting that PET/CT could improve survival outcome because of an enhanced ability for detecting bone metastases.

Imaging follow-up is designed to detect postsurgical recurrences. Recurrent bone sarcomas are entirely curable as long as lesion resection is possible.^65^ Because of post-treatment changes and image artifacts caused by metallic endoprostheses, the detection of local recurrence using traditional anatomic modalities has been shown to be inferior to functional imaging.^66,67^ We found that ^18^F-FDG PET/CT had good accuracy for the detecting bone sarcoma recurrence, which was similar to that noted for other recurrent malignancies.^12,68,69^

The histological response to chemotherapy, number and sites of distant metastatic lesions, and local recurrence are all significant prognostic indicators. However, radical resection of metastatic lesions significantly improves survival.^70^ Therefore, accurate staging, restaging, and recurrence surveillance of bone sarcomas by ^18^F-FDG PET and PET/CT could provide information for risk stratification that could eventually translate into a clinical survival benefit.

Although satisfactory results have been demonstrated, considering the mechanism of ^18^F-FDG PET and PET/CT, FP and FN cases are unavoidable. There are multiple factors affecting the possibility of a misdiagnosis. First, some aggressive benign tumors (such as giant cell tumor of the bone) and inflammatory lesions^71^ are ^18^F-FDG-avid, with the inflammatory lesions being responsible for the majority of FP cases. Second, not all bone sarcoma types can be definitively identified according to ^18^F-FDG uptake, for example, chondrosarcoma shows only low or moderate ^18^F-FDG uptake.^29,32,36^ Third, nonspecific ^18^F-FDG uptake and asymmetric ^18^F-FDG distribution in malignant diseases can complicate the interpretation for radiologists. Morphologic information acquired by the CT portion of PET/CT partially compensates for the deficiencies in ^18^F-FDG uptake in a small proportion of bone sarcomas, therefore improving diagnostic accuracy. However, as mentioned above, because of the limitations of CT, some subcentimeter lesions may still be missed. Therefore, the findings of ^18^F-FDG PET and PET-CT in bone sarcomas should be confirmed by a histopathological examination or follow-up.

Besides inherent limitations of meta-analysis such as publication and selection bias, there are some limitations to the present study. First, the proportions of sarcoma subtypes in retrieved trials varied. Because of the low incidence of primary bone sarcoma, detailed and homogeneous analysis based on sarcoma subtype was not possible. Consequently, underestimations or overestimations might exist in the present data. Second, multiple methods to measure ^18^F-FDG avidity and multiple cut-offs to determine lesion positivity, as well as multiple other study factors, were employed across different studies. Third, the patients’ characteristics information was incomplete in some studies. Although we tried to obtain comprehensive information from the authors of original papers, some data remained unavailable. Fourth, several subgroup analyses were based on a small number of studies or were not possible because of incomplete data; especially for ^18^F-FDG PET, which could reduce the power of our statistical analyses.

## CONCLUSION

This systemic review of the published literature demonstrated that ^18^F-FDG PET and PET/CT could be applied to differentiate primary bone sarcomas from benign lesions. Moreover, PET/CT was useful for the diagnosis, staging, restaging, and recurrence surveillance of bone sarcomas, although a relatively low sensitivity at detecting lung metastatic lesions was observed. Nevertheless, the possible existence of FP and FN cases merits consideration. Pathological examination or long-term follow-up should be carried out for ^18^F-FDG-avid lesions in patients with bone sarcomas.
